# Editorial: Therapeutic potential of natural products in oxidative and metabolic diseases

**DOI:** 10.3389/fphar.2024.1375788

**Published:** 2024-02-23

**Authors:** Aliyu Muhammad, Chika Ifeanyi Chukwuma, Ochuko Lucky Erukainure, Md. Shahidul Islam

**Affiliations:** ^1^ Department of Biochemistry, Faculty of Life Sciences, Ahmadu Bello University, Zaria, Kaduna, Nigeria; ^2^ Centre for Quality of Health and Living (CQHL), Faculty of Health and Environmental Sciences, Central University of Technology, Bloemfontein, Free State, South Africa; ^3^ Department of Microbiology, School of Life Sciences, University of KwaZulu-Natal, Durban, South Africa; ^4^ Department of Biochemistry, School of Life Sciences, University of KwaZulu-Natal, Durban, South Africa

**Keywords:** phytochemicals, functional foods, oxidative stress, diabetes, obesity, cardiovascular diseases

## Introduction

Research on the therapeutic potential of natural products in oxidative and metabolic diseases has gained significant attention in the recent years. Oxidative stress and metabolic dysregulations have been implicated in various health conditions, including cardiovascular diseases, diabetes, neurodegenerative disorders, obesity and so on (Salau et al.). Natural products, derived from plants are rich in bioactive compounds with antioxidant and metabolic modulating properties (Chanu et al.). This thematic issue was conceived on the baseline information that oxidative and metabolic diseases represent a large proportion of global public health challenges and quality of life. These diseases are often characterised by the imbalance between the cellular prooxidant (products of metabolic processes) and the antioxidant molecules within the cells. The imbalance often leads to the underlying factors that exacerbate the pathogenesis of some life-threatening diseases such as cancer, obesity, diabetes, and cardiovascular diseases. On the other hand, natural products, including phytochemicals and functional foods are known for their ability to modulate metabolic processes such as ameliorating reactive oxygen species (ROS)-induced mitochondria dysfunction, mitigating inflammatory response, and other cellular functions that could ameliorate the disease developments (Alruhaimi et al.; Chanu et al.). Despite the relatively low toxicities of natural products as compared to synthetic medicines, further studies are still required to optimize their bioavailability, therapeutics, pharmaceutics, at different experimental settings including pre-clinical and clinical trials in patients suffering from oxidative and metabolic diseases.

Over the years, the treatment and management of metabolic disorders and metabolic syndromes including diabetes, obesity and cardiovascular diseases have been challenging due to higher cost, lack of safety and several adverse effects of the synthetic drugs (Chanu et al.). As these disorders are exacerbated by oxidative and inflammatory stress, natural products including phytochemicals, functional foods, and nutraceuticals with antioxidative and anti-inflammatory properties have now been increasingly utilized as an alternative therapeutics. Natural products are naturally embedded with pharmacological activities useful for the prevention and treatment of various diseases. They are often utilized as starting points for drug discovery research which can lead to the development of a new drug with improved efficacy and safety. Metabolic disorders are multifactorial diseases, thus the use of natural products as a supplement may also be effective in the treatment of these diseases.

The envisaged Research Topics are a collection of quality original research articles that provide both experimental and clinical data on the potential therapeutic relevance of natural products, including phytochemicals and functional foods in the management of oxidative and metabolic diseases. In addition to original research articles, we were able to accommodate some well-articulated review articles with original interpretations of existing knowledge to expose scientific gaps and to provide new insights into the therapeutic prospects of phytochemicals and functional food in oxidative and metabolic disorders. The thematic areas which were relevant to the therapeutic relevance of natural products in diabetes and obesity, cardiovascular diseases, and oxidative stress and inflammation were considered in the Research Topic. In this editorial, we attempted to highlight the findings and potential impacts of some of the notable articles published under the Research Topic as briefly described below.

As shown in some of the studies, the antidiabetic potentials of natural products comprise different mechanisms. Chanu et al.’s study provided scientific support for the utilization of *Ageratina adenophora* in treating type 2 diabetes in a the streptozotocin (STZ) and nicotinamide (NA)STZ-NA-induced diabetic rat model. The possible mechanisms include inhibiting carbohydrate digestion and improving insulin sensitivity. Alruhaimi et al. demonstrated that a flavonoid-rich fraction of *Euphorbia peplus* attenuates hyperglycaemia, insulin resistance, and oxidative stress in a type 2 diabetes rat model. Berberine’s ability to enhance the function of the β-cells of pancreatic islets in db/db mice through the GLP-1/GLP-1R/PKA signalling pathway was reported by Wu et al. Salemcity et al. demonstrated the reversal of mitochondrial permeability transition pore and pancreas degeneration by the chloroform fraction of *Ocimum gratissimum* (L.) leaf extract in a type 2 diabetic rat model. All the above-mentioned studies highlighted the potentials of natural products and compounds for the management of type 2 diabetes.

Some other studies suggest that the anti-inflammatory and antioxidant potentials of natural products could mitigate the damage of key metabolic organs and improve associated disease conditions. Alshehri and Alorfi elucidated the protective efficacy of resveratrol against Vancomycin-induced hepatotoxicity in male Wistar rats through oral administration. The co-treatment of resveratrol with vancomycin demonstrated a pronounced hepatoprotective effect, averting the elevation of key markers such as AST, ALT, ALP, IL-6, and MDA. Additionally, it protected the liver from the depletion of NO and GSH. Salau et al. documented the hepatoprotective effects of *Lippia javanica* (Burm. F.) Herbal Tea in Chang liver cells by mitigating redox imbalance and perturbed metabolic activities. This underscores its potential as a therapeutic agent for oxidative stress-related liver diseases.

Abo-Saif et al. reported the cardioprotective potential of pomegranate peel extract in diabetic rats, attributing its efficacy to unique antioxidant, anti-inflammatory, and antifibrotic properties. The inhibition of the NLRP3/caspase-1/IL-1β signalling pathway and downregulation of lncRNA-MALAT1 were identified as key mechanisms in this study. The study of Mazhar et al. showed that Zhilong Huoxue Tongyu capsule could attenuate intracerebral haemorrhage-induced redox imbalance by modulating the Nrf2 signalling pathway.

The therapeutic potential of natural products on dyslipidaemia was also documented. Abduh et al. established that *Averrhoa carambola* leaves effectively prevent dyslipidaemia and oxidative stress in a rat model of acute hyperlipidaemia-induced by poloxamer-407. This protective effect was attributed to the modulation of factors related to lipoprotein lipase (LPL), phospholipids (PL), HMG-CoA reductase, and cholesterol synthesis. Xin et al. reported that *Polygala tenuifolia* Willd. seed oil (PWSO) treatment inactivated SREBP1 and SREBP2, inhibiting hepatic lipid accumulation and mitigating inflammation via the NF-κB signalling pathway. This study suggested the potential use of PWSO as a dietary supplement to inhibit the occurrence and development of metabolism-associated fatty liver disease (MAFLD).

Sayed et al.’s comprehensive review provided insights into the mechanisms of action and therapeutic potential of natural products as novel anti-obesity agents. In another review, Wang and Li reported the therapeutic role of baicalin in various lung diseases, including chronic obstructive pulmonary disease, asthma, pulmonary fibrosis, pulmonary hypertension, pulmonary infections, acute lung injury/acute respiratory distress syndrome, and lung cancer, while elucidating the underlying mechanisms.

Overall, these insights encourage continued exploration of natural products for their valuable contributions to preventive and therapeutic strategies in human health, particularly for oxidative and metabolic disorders.

